# Antimicrobial Activity of New Materials Based on Lavender and Basil Essential Oils and Hydroxyapatite

**DOI:** 10.3390/nano8050291

**Published:** 2018-04-30

**Authors:** Daniela Predoi, Simona Liliana Iconaru, Nicolas Buton, Monica Luminita Badea, Luminita Marutescu

**Affiliations:** 1 National Institute of Materials Physics, 405 A Atomistilor Street, P.O. Box MG7, 077125 Magurele, Romania; simonaiconaru@gmail.com; 2 HORIBA Jobin Yvon S.A.S., 6-18, Rue du Canal, 91165 Longjumeau CEDEX, France; nicolas.buton@horiba.com; 3 University of Agronomic Sciences and Veterinary Medicine, Faculty of Horticulture, 59 Mărăşti Blvd., 011464 Bucharest, Romania; badea.artemisia@gmail.com; 4 Microbiology Department, Faculty of Biology, University of Bucharest, 1–3 Portocalelor Lane, 77206 Bucharest, Romania; lumi.marutescu@gmail.com; 5 Earth, Environmental and Life Sciences Section, Research Institute of the University of Bucharest (ICUB), 91-95 Splaiul Independentei, 050095 Bucharest, Romania

**Keywords:** hydroxyapatite, essential oil, Dynamic Light Scattering (DLS), antimicrobial studies

## Abstract

This study presents, for the first-time, the results of a study on the hydrodynamic diameter of essential oils (EOs) of basil and lavender in water, and solutions of EOs of basil (B) and lavender (L) and hydroxyapatite (HAp). The possible influence of basil and lavender EOs on the size of hydroxyapatite nanoparticles was analyzed by Scanning Electron Microscopy (SEM). We also investigated the in vitro antimicrobial activity of plant EOs and plant EOs hydroxyapatite respectively, against Gram-positive bacteria (methicillin-resistant *Staphylococcus aureus*1144 (MRSA 1144) and *S. aureus* 1426) and Gram-negative bacteria (*Escherichia coli* ATCC 25922 and *Escherichia coli* ESBL 4493). From the autocorrelation function, obtained by Dynamic Light Scattering (DLS) measurements it was observed that basil yielded one peak at an average hydrodynamic diameter of 354.16 nm, while lavender yielded one peak at an average hydrodynamic diameter of 259.76 nm. In the case of HAp nanoparticles coated with basil (HApB) and lavender (HApL) essential oil, the aggregation was minimal. We found that the lavender EO exhibited a very good inhibitory growth activity (MIC values ranging from <0.1% for *E. coli* reference strain to 0.78% for *S. aureus* strains). The biological studies indicated that HapL material displayed an enhanced antimicrobial activity, indicating the potential use of HAp as vehicle for low concentrations of lavender EO with antibacterial properties. Flow cytometry analysis (FCM) allowed us to determine some of the potential mechanisms of the antimicrobial activities of EOs, suggesting that lavender EO was active against *E. coli* by interfering with membrane potential, the membrane depolarization effect being increased by incorporation of the EOs into the microporous structure of HAp. These findings could contribute to the development of new antimicrobial agents that are urgently needed for combating the antibiotic resistance phenomena.

## 1. Introduction

Recent advances in medicine and biotechnology have not been able to overcome the rapid and ongoing worldwide emergence and spread of drug resistant microorganisms. This fact has stimulated the search for new types of effective and nontoxic antimicrobial agents amongst natural compounds which are found in aromatic plants, and which have been previously used in cosmetics, folk medicine, and aromatherapy [1]. Nowadays, many plants possess great economic value, and it is estimated that approximately 700,000 species of tropical plants exhibit medicinal properties and can be used as antibacterial, antiviral, antifungal, antiallergic, anticarcinogenic and larvicidal agents [2,3]. Essential oils, which have been known for their perfume, flavor and preservative properties since antiquity, are aromatic oily liquids and can be obtained from different plant organs (flowers, buds, seeds, leaves, twigs, etc.) by expression, extraction, fermentation, enfleurage, and steam distillation [4,5]. It has been reported several times that some EOs possess antimicrobial properties [5,6,7,8], and due to the recent worldwide interest in “green” consumerism, the scientific interest regarding these substances has been revitalized [9,10]. Beyond their antibacterial properties [11], EOs have also been reported to exhibit antiviral [12], antimycotic [13], antioxigenic [14], antiparasitic [15], and insecticidal [16] properties. 

The bactericidal properties of EOs vapors are presumed to have been investigated for the first time in 1881 by De la Croix [9]. Nevertheless, the use of EOs in medicine has become secondary to their uses as flavors and aromas in the 19th and 20th centuries [5]. Nowadays, the antibacterial properties of EOs and their components are exploited mostly in commercial products as dental root canal sealers [17], cosmetic preservatives [18], antiseptics [19], food packaging [20,21,22], etc. Although currently used in many commercial products due to their aromatic properties, EOs are widely known for their role in food safety and preservation [23,24,25,26,27,28]. Due to its biological properties and attractive aroma, lavender EO is widely used in the food industry [29,30,31,32,33]. Recent studies have reported the use of lavender in food industry for extending the quality and shelf life of bread [34]. Moreover, in their studies, Bensmira et al. have demonstrated that the incorporation of lavender oil into sunflower oil has a promising effected on the frying temperature of the oil [35]. In particular, it has been reported to demonstrate antimicrobial properties against a variety of microorganisms, especially *Candida albicans*, *Escherichia coli* and *Staphylococcus aureus* [36,37]. Due to its outstanding antimicrobial properties, lavender EO has also been investigated for use in strawberry packaging systems [38]. Sangsuwan et al. [38] demonstrated in their study that the overall quality of the fruit treated with lavender and red thyme EOs were maintained. Beyond that, the results revealed that lavender EO can extend the mold-free storage life of strawberries kept at 7 °C from 2 days (control) to 8 days. Over recent decades, anecdotal evidence has described the biologic activity of lavender EOs, but it is only recently that some of the claims have been scientifically investigated [37,39]. Another popular herb, basil, often referred as the “King of the herbs”, which belongs to the major oil-producing species, is widely utilized due to its economic, nutritional, industrial and medicinal importance [40]. Basil EO has been reported to exhibit antioxidant, antimicrobial and antitumor activities, due to its phenolic acids and aromatic compounds [41,42]. Recent studies have reported that different Eos, including those of lavender and basil, have antibacterial activities against gram positive bacteria, including *Staphylococcus aureus*, *Bacillus* species, and gram negative bacteria, namely *Escherichia coli*, *Shigella flexneri*, as well as pathogenic fungus, specifically *Candida albicans* [43,44,45,46]. Two of the most prevalent species of gram-positive and gram-negative bacteria that are responsible for very severe infections are *Staphylococcus aureus* and *Escherichia coli*. *S. aureus* is one of the most common Gram-positive organisms, and is responsible for a multitude of diseases, ranging from moderate skin infections and food poisoning to endocarditis and very severe bloodstream infections. *E. coli* is also a common nosocomial pathogen that causes urinary tract infections and enterocolitis. According to data from the Broad Institute in 2010, *E. coli* was responsible for 17.3% of of clinical infections requiring hospitalization, while *S. aureus* was accountable for 18.8% [47,48]. Due to the worldwide emergence of *S. aureus E. coli* strains which are resistant to conventional antibiotic therapy, there have been major concerns in the public health area invigorating the need for the development of new antimicrobial compounds. Hydroxyapatite is one of the most commonly materials used in the pharmaceutical and medical fields, due to its similarities to the human hard tissue, and its biocompatible properties. HAp has been utilized in biomaterial engineering and regenerative medicine, and was employed in the development of controlled drug delivery systems [49,50,51]. Besides its use in the medical field, HAp crystals are well known for their use in the food industry and agriculture. Initially HAp was utilized for softening drinking water, but after 1985, due to its remarkable properties for removing pollutants from water, HAp has been used for metal and nutrient recovery from wastewater systems [52,53]. Owing to its similarities with hard human tissue, HAp is currently being widely investigated for its possible biomedical applications as an antimicrobial agent. So far, studies have been undertaken involving HAp powders doped with several metal ions that are known to possess antimicrobial properties, such as silver, zinc, cerium [54,55,56,57], etc. In this context, the present work is focused on the use of hydroxyapatite as a carrier of Eos, in order to develop a new antimicrobial bioactive compound. 

In this work, studies regarding the hydrodynamic diameters of basil and lavender EOs in water and of HAp nanoparticles coated with basil and lavender EOs (HApB and HApL) are presented for the first time. The antimicrobial activity of the newly-developed materials based on HAp nanoparticles (HAp-B and HAp-L) and plant EOs (B-Basil and L-Lavender) against Gram-negative (*E. coli*) and Gram-positive (*S. aureus*) bacteria was also investigated.

## 2. Results and Discussions

Both *Ocimum basilicum* L. (basil or sweet basil) and *Lavandula angustifolia Mill* (lavender) are some of the world’s most cultivated varieties, due to their various applications in the food, cosmetics, and perfume industries, as well as their use in traditional medicine [58,59].

The most abundant components identified in basil and lavender EOs are presented in Table 1 and Table 2. 

The high concentration of linalool in the samples indicates that basil EO is of chemotype A. The GC data emphasized that the majority of the compounds found in the samples were monoterpenes and sesquiterpenes. The results of the analysis revealed that the dominant components of the lavender EO samples were represented by the oxygenated monoterpenes group. In both the basil and lavender EOs GC/GCMS analyses, the rest of the identified constituents were present at under 0.5%; numerous compounds were identified as trace amounts. Although plant EOs have been utilized for over 6000 years, scientific research of them is currently limited to fundamental, phytochemical and biological research. The physico-chemical properties of plant EOs are not well understood. Their ability to self-aggregate in solution is also not completely understood.

Recently, particular attention has been paid to HAp as a result of the widening of application domains, such as its use in the preparation of biocompatible materials for dental and orthopedic applications, or use in the manufacture of pharmaceutical products in the food or fertilizer industries [60]. Structure, crystallinity, particle size or morphology play an important role in each of these applications. This research was focused on studying the structure and morphology of materials based on HAp and EOs. In this context, information on the structure and morphology of HAp synthesized prior to EOs coverage could be of particular importance in various applications in the pharmaceutical or food industries.

**Figure 1:**
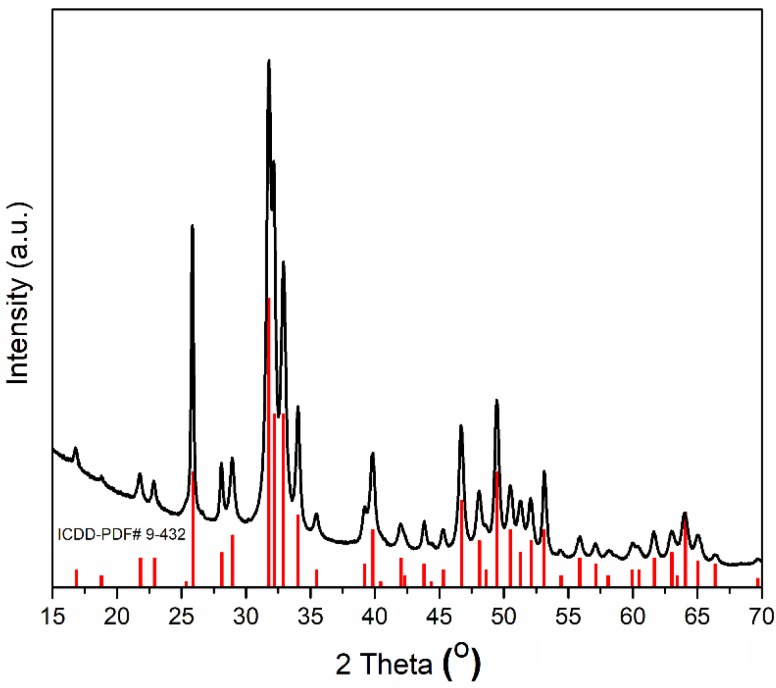
The X-ray diffraction (XRD) patterns of HAp (ICDD-PDF No. 9-432) and obtained HAp powders after centrifugation and drying at 100 °C in an oven.

The X-ray diffraction (XRD) patterns of the obtained HAp powders, after centrifugation and drying at 100 °C in an oven, are shown in Figure 1. XRD results of HAp revealed that the peaks were perfectly matched with the standard ASTM data ICDD-PDF No. 9-432. All the peaks were assigned to the hexagonal Ca_10_(PO_4_)_6_(OH)_2_ in P63/m space group. The peaks of impurities, such as calcium hydroxide and calcium phosphates were not observed (Figure 1). The calculated values of lattice parameters “a” and “c” were 9.424 and 6.8839 Å, while for reference HAp (ICDD-PDF No. 9-432), the lattice parameters “a” and “c” were 9.418 and 6.8840 Å. The crystallite size of the prepared HAp obtained from Scherrer’s equation was 83.5 ± 1 nm. Similar results were also obtained in our previous studies [61,62]. According to A. Bigi et al. [62], the values of the parameters of the sides “a” and “c” are influenced both by the synthesis method, and the pH at which synthesis was achieved.

**Figure 2:**
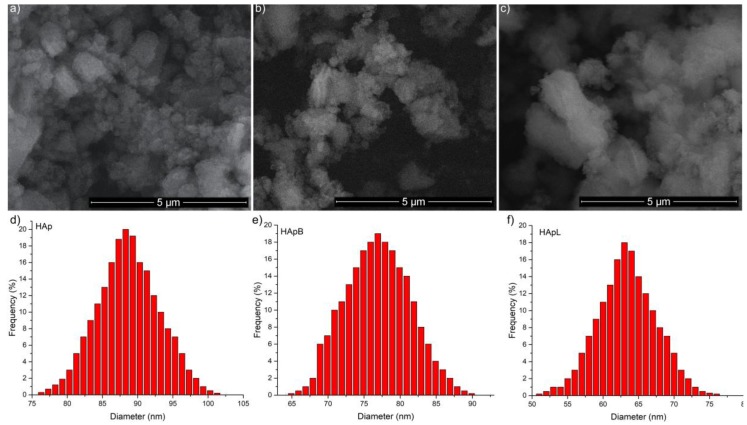
Scanning Eelectron Microscopy (SEM) images of the HAp (**a**), HApB (**b**) and HApL (**c**) samples. Size distributions of HAp (**e**), HApB (**f**) and HApL (**g**) samples.

The morphology of the newly developed materials based on HAp and basil and lavender EOs (HApB and HApL) were studied using SEM. The influence of basil and lavender EOs on the size of HAp nanoparticles is presented in Figure 2a–c. The surface morphology did not change after the EO adsorption on the surface of the HAp nanoparticles. From the Scanning Electron Microscopy (SEM) images of the samples, the average size of the particles were determined by counting the number of particles (around 200). The particle distribution of HAp, HApB and HApL is presented in Figure 2d–f. 

SEM images revealed that the particles were nanosized (less than 100 nm), with the size of nanoparticles decreasing from HAp to HApB and HApL. The mean particle sizes of HApB and HApL samples were evaluated at 76.8 ± 5 nm and 63.3 ± 6 nm, while the mean particle size of HAp was estimated to be 88.5 ± 3 nm. 

Moreover, studies regarding the hydrodynamic behavior of HAp nanoparticles coated with basil and lavender EOs were also carried out. According to J. Bodycomb [63], the random fluctuations are interpreted in terms of the autocorrelation function (ACF), *C*(τ):

*C*(τ) = 1 + βexp(−2Γτ)

where the decay constant is Γ = *D*_m_*q*^2^ and *q* = (4πn/λ)sin(θ/2), with *D*_m_ diffusion coefficient at infinite dilution, *q* scattering vector, *n* refractive index, λ wavelength, and θ scattering angle. 

Finally, the hydrodynamic diameter obtained is the diameter of a sphere having the same translational diffusion coefficient as the particle. The hydrodynamic diameter was calculated using the Stokes-Einstein equation [54]:

*D*_h_ = k_B_*T*/3πƞ*D*_m_

where *D*_h_ is the hydrodynamic diameter, k_B_ the Boltzmann constant, T absolute temperature and ƞ the viscosity of the solvent. 

**Figure 3:**
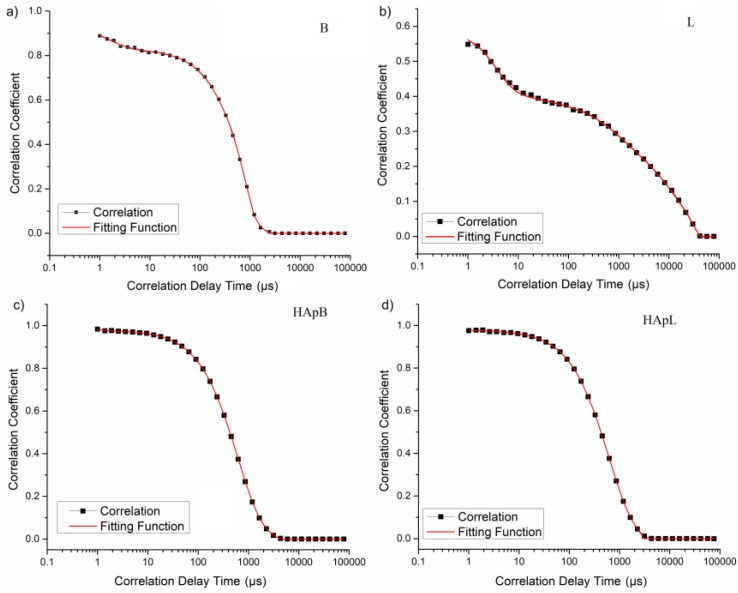
Experimental (markers) and fitted (red line) autocorrelation function obtained with DLS experiments for B (**a**); L (**b**); HApB (**c**) and HApL (**d**) samples.

An autocorrelation function of the scattered signal was obtained for all the studied samples (Figure 3). 

Figure 3 revealed the experimental (markers and black line) and fitted (red line) autocorrelation function, obtained by DLS experiments on the basil and lavender EOs in water (Figure 3a,b) and HA coated with basil and lavender EOs in water (Figure 3c,d). A low scattering intensity was detected for lavender EOs in water (Figure 3b).

**Figure 4:**
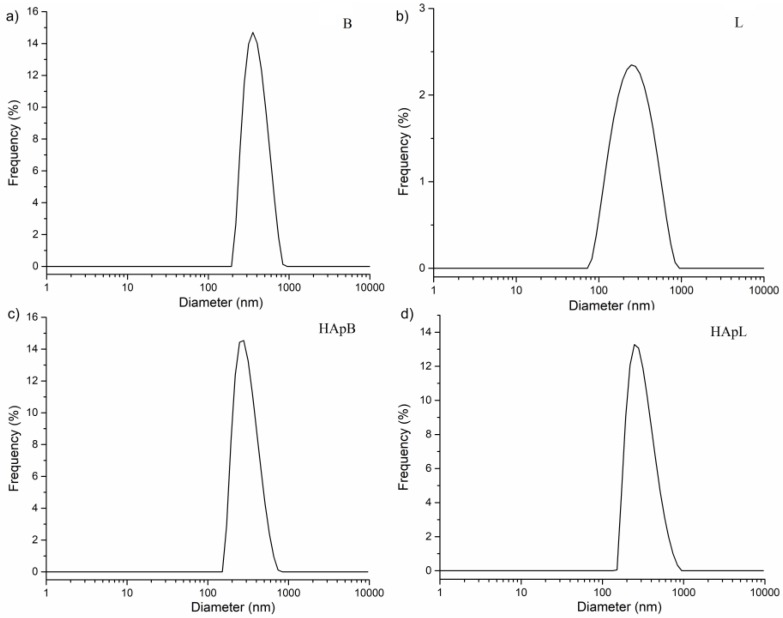
Particle size distributions of basil B (**a**); L (**b**); HApB (**c**) and HApL (**d**) obtained by fitting the experimental autocorrelation function.

In Figure 4, the histogram obtained from the regularization fit of the autocorrelation was illustrated. It was observed from the autocorrelation function that basil yielded one peak at an average hydrodynamic diameter of 354.16 nm, while lavender yielded one peak at an average hydrodynamic diameter of 259.76 nm.

For the HApB and HApL samples, the average hydrodynamic diameters were established to be 265.63 and 257.76 nm respectively. The comparison of mean particle size estimated from SEM and DLS analysis is presented in Table 3. The difference between the particle diameters determined by the two methods is due to the fact that while SEM uses a primary electrons beam that is scanned over the surface of a specimen, whereas the Dynamic Light Scattering (DLS) measures the diffusion coefficient of the translational of the particles moving randomly in a liquid medium by Brownian motion. The diffusion coefficient is inversely proportional to the hydrodynamic diameter of the particles by the Stokes-Einstein equation [54].

The mean hydrodynamic diameter value of HAp was 273.86 ± 2 nm. The values obtained by SEM (88.5 ± 3) and XRD (83.5 ± 1 nm) analysis for mean diameter were considerably smaller than those obtained by DLS. Since there is a very large difference between the diameters obtained by SEM, XRD and DLS, this difference cannot be explained by taking into account the solvation layer of the particles. This behavior suggests the formation of suspension agglomerates. This mechanism could be explained as a result of the relative strength of hydrogen bonds that determine the formation of superaggregates, which immediately precipitate in the dispersion. It should be highlighted that all the samples demonstrated the same behavior.

The results obtained from DLS measurements in this study for all the analyzed samples are in good agreement with those previously presented by Lucio et al. [64]. Moreover, Wang et al. [65], in their recent studies regarding β-cyclodextrin and modified magnetic graphene oxide nanocomposites, confirmed our results. On the other hand, the materials based on HAp coated with basil and lavender EOs exhibited similar behavior to that of the β-cyclodextrin/HAp composites presented by Predoi et al. [66] in recent studies.

As a result of the increase in the incidence of antibiotic-resistant pathogens, particular attention was paid to the microstatic and microcidal actions, as well as to the spectrum of affected organisms. Exploitation of EOs obtained from medicinal plants for the treatment of microbial infections can lead to the development of new materials with antimicrobial properties. Since EOs are traditionally mainly used in aromatherapy [67] and massage [67], in the present study we have proposed the investigation of the antimicrobial activity of EOs in water solutions and EOs HAp solutions. 

In the current context, when more and more bacterial strains adapt to new living conditions and develop an increased resistance to antibiotics, our intention to find and develop new materials based on EOs capable of stopping the development of bacteria, and even killing them, is an important step in this area. 

The effect of plant EOs (basil and lavender) and HApB and HApL samples on cell viability was appraised against gram-positive (MRSA 1144 and *S. aureus* 1426) and gram-negative (*E. coli* ATCC 25922 and *E. coli* ESBL 4493) bacteria. One of the objectives of this research was to obtain significant information about the antibacterial effects of plant EOs, HApB and HApL. The qualitative screening of the antimicrobial properties was performed using an adapted diffusion method. The inhibition zone diameter results are presented in Table 4. The lavender EO inhibited the growth of all the tested bacterial strains, as indicated by the formation of growth inhibition zones around that ranged from 16 mm (against *E. coli* ESBL 4493) to 24 mm (against MRSA 1144). The HApL material was active against the tested bacterial strains, when compared to HAp alone. On the other hand, the basil EO and the HApB samples exhibited a lower inhibitory effect against the tested bacteria. The HAp materials had no effect on the growth of the selected bacteria. 

Quantification of the antimicrobial activities of plant EOs and plant EO coated HAp was carried out using the microdilution method. The minimum inhibitory concentration (MIC) and values of the minimum bactericidal concentration were determined using the conventional plating method. The results are presented in Table 5 and Table 6.

The lavender EO displayed a stronger antimicrobial activity against the selected bacterial strains compared to the basil EO [68,69]. The Gram-negative bacteria, *E. coli* ATCC 25922 and *E. coli* ESBL 4493 were the most susceptible, with MBC values 0.19% for *E. coli* ESBL 4493 and of 0.1% for *E. coli* ATCC 25922 indicating a strong antimicrobial activity of lavender EO. The tested Gram-positive bacteria were less susceptible to the antibacterial action of the lavender EO. A possible explanation is that Gram-positive bacteria harbor a thick layer of peptidoglycan, which has the potential to inhibit the membrane-disrupting action of many of the EOs [68,69]. 

HAp alone lacked a noticeable antibacterial action, since the MBC values recorded against all bacteria were equal or greater than 5 mg/mL. However, significantly increased antimicrobial activity was detected for the HAp coated with lavender EO (HApL) against all the tested bacteria, as demonstrated by lower MIC values (ranging from 0.15 mg/mL in case of *E. coli* ATCC 25922 to 0.62 mg/mL (*E. coli* ESBL 4493). These results suggest that hydroxyapatite nanoparticles can be used as carriers for low concentration of lavender EO with antibacterial properties. Basil EO exhibited a lower antimicrobial effect in comparison with lavender EO; the recorded MIC and MBC values against the tested bacteria ranging between 25 and 50%. Also, the results indicated minor changes regarding the biological activity of HApB against the tested bacteria.

The flow cytometry (FCM)measurements were carried out for interrogation of the membrane potential of microbial cells treated with basil and lavender Eos, and the newly developed materials respectively, at ½ × MICs, using oxonol DiBAC_4_(3), as an indicator for membrane depolarization. The dye accumulates in the cytoplasm of depolarized bacteria and binds to intracellular proteins or membranes, thus resulting in an enhanced green fluorescence [70].

**Figure 5:**
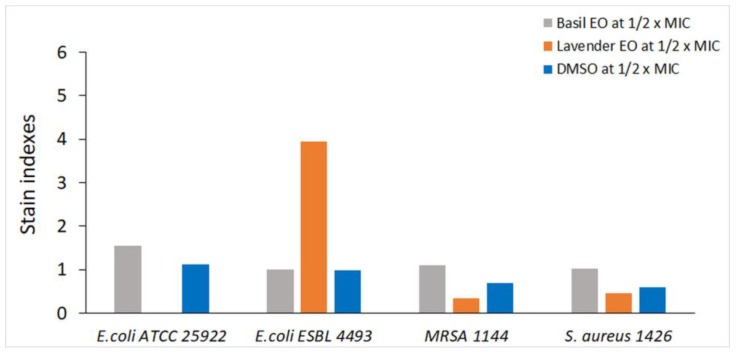
Stain indexes for each tested strain. The stain index is the ratio of the intensity of fluorescence measured in the FITC channel (530/30 nm), of bacteria treated with plant EO and DMSO respectively to that of untreated cells.

The intensity of the fluorescence (IF) of the bacterial cells treated with plant EOs alone were compared and are presented in Figure 5. The IF of the bacterial strains exposed to the plant EOs were similar with those of dimethyl sulfoxide (DMSO) treated cells, with the exception of *E. coli* ESBL 4493 cells treated with lavender EO. We observed a strong depolarization of bacterial plasmatic membrane, indicated by an increase in IF of at least three times in lavender EO treated cells compared with the solvent treated cells, at ½ × MIC. These results suggested that lavender EO was active against *E. coli* ESBL 4493 by interfering with the membrane potential. The basil EO caused a slight increase in IF, at ½ × MIC, in the case of *E. coli* ATCC 25922, MRSA1144 and *S. aureus* 1426, suggesting other possible antimicrobial mechanisms of action for this plant EO.

**Figure 6:**
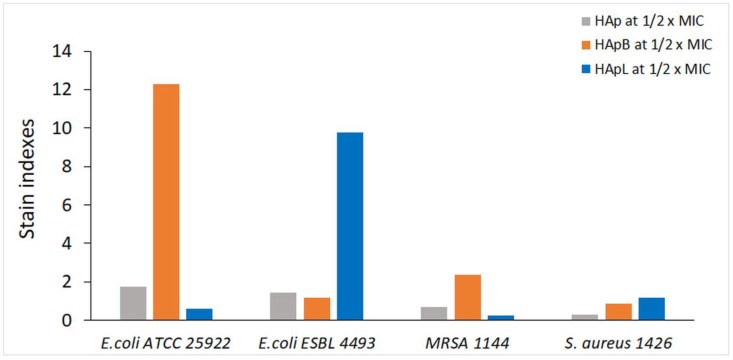
Stain indexes for each tested strain. The stain index is the ratio of the intensity of fluorescence measured in the FITC channel (530/30 nm), of bacteria treated with HAp, HApB and HApL to that of untreated cells.

**Figure 7:**
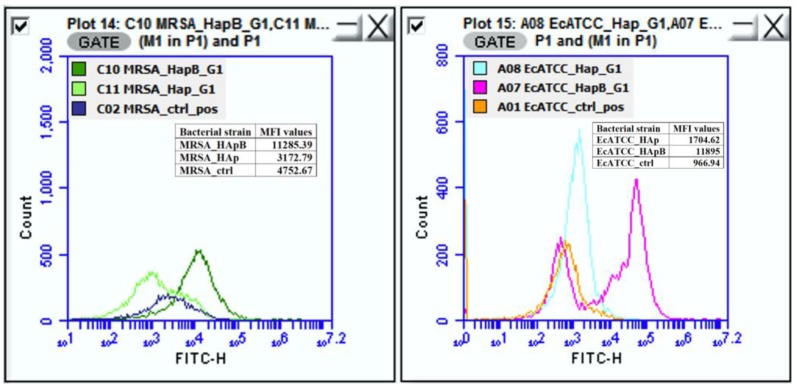
Overlays of control populations (not treated) onto exposed cells to HAp alone and HApB, respectively.

We found that the plasmatic membrane depolarization effect was increased by the incorporation of the basil EO into the structure of HAp for *E. coli* ATCC 25922 and MRSA 1144, as indicated by the increase of at least two times in the IF, when compared with HAp alone (Figure 6 and Figure 7). These results suggested stronger antimicrobial activity in the case of the newly developed materials. However, this effect was not detected against *E. coli* ESBL 4493 and *S. aureus*1426, when compared with HAp alone. 

The incorporation of lavender EO in HAp did not interfere with the antimicrobial mode of action of the lavender EO, as indicated by the loss of membrane potential of *E. coli* ESBL 4493 treated cells, when comparing with HAp treated cells. No significant differences were detected for the *S. aureus* 1426 bacterial strain treated with HApL and HApB, when compared with the HAp alone, suggesting other possible antimicrobial mechanisms.

Lavender and basil EOs have been known and used since ancient times both in Greece and ancient Rome, as well as in the mysterious ancient Egypt [71,72,73,74], for perfume and medicine. Hippocrates and Theophrastus [75] talked about their beneficial effects on general state and mood. As claimed by T. Moon et al. [76] in their studies on “antiparasitic activity of two *Lavandula* essential oils against *Giardia duodenalis*, *Trichomonas vaginalis* and *Hexamita inflata*”, lavender was one of the holy herbs used in the biblical Temple to prepare the holy essence. According to M. Lis-Balchin [77], lavender was also mentioned in Solomon’s song. The recent studies conducted by LG Matasyoh et al. [78] revealed that various plant EOs possess antibacterial activities against different gram negative (*Escherichia coli*, *Salmonella enterica* serotype *Enteritidis*) and gram positive bacteria (*Staphylococcus aureus*, *Bacillus* species), and fungi such as *Candida albicans*. In their previous studies, S. Dube et al. [79] reported the antifungal, physicochemical, and insect-repelling activity of the EO of *Ocimum basilicum*. Previous studies conducted by L. L. Silver and his collaborators [80] have shown increasing interest among researchers to discover and develop new antibiotics from medicinal plants.

In recent years, as a result of the increase in the number of drug resistant pathogens, special attention has been paid to the development of new antimicrobial agents. Plant EOs have been used since ancient times as natural remedies to combat pathogens. The antimicrobial action mechanisms include damaging the cell wall and membrane, alteration of proton motive force, and leakage of cytoplasmic contents [81]. Several approaches have been reported using plant EOs or components for prevention and control of human and animal infections. Also, many promising applications, such as pharmaceutical, food preservation systems, crop protection, are emerging [82]. However, toxicity, volatility, and lack of stability are currently limiting the transfer from research to practice. Antimicrobial coating has many advantages, including: efficient release of drugs, elevated stability, and protection from the interactions with the environment, decrease in volatility, enhancement of bioactivity, and reduced toxicity. In this context, we developed novel antimicrobial agents based on the incorporation of lavender and basil essential EOs into HAp. Our results regarding the antimicrobial activity of basil EO and of the newly-obtained material based on HAp and basil EO are in agreement with those previously reported by G. K. Sinha and B. C. Gulati [83]. In prior studies [84] concerning the antimicrobial and antifungal activity of plant EOs and some of their constituents, basil EOs were effective against a wide range of bacteria (e.g. *S. aureus*, *E. coli*, *Salmonella enterica* serotypes *typhi* and *paratyphi*, *Shigella boydii* and *Proteus vulgaris*).

The data presented in this paper regarding the antibacterial activity of lavender EO and newly developed lavender EO coated HAp are consistent with previous studies conducted by M. Lis-Balchin et al. [85,86] which have shown that lavender EO is active against many species of bacteria and fungi. The findings of this study also confirmed the previous research carried out by R. R. S. Nelson [87] on the in vitro activity of lavender essential oil against methicillin-resistant *Staphylococcus aureus* and vancomycin-resistant *Enterococcus faecium*, at very low MIC values (less than 1%). Moreover, in the present study, we have demonstrated that the newly developed material based on HAp and lavender EO exhibits a strong antimicrobial property against representative Gram negative and Gram positive bacteria. In a recent study [88], the most effective calcium hydroxyapatite has been shown to be the microcrystalline hydroxyapatite concentrate extracted from the bones and or bone marrow of cattle, due to high protein and vitamin C levels. However, there is a risk that this calcium hydroxyapatite will be contaminated with various heavy metals such as lead, arsenic, or cadmium, from the animals feed. Moreover, as they come from cattle, there is the risk that the alfalfa, corn, or soybean have been genetically modified. As a result, the obtaining of hydroxyapatite by a simple and inexpensive method that can control purity [55,56,66] presents a particular advantage for the food industry. The possibility of covering hydroxyapatite with different molecules (such as EO) makes it more appealing to the food industry, and its range of uses could increase significantly. Moreover, starting from research on hydroxyapatite, the “nanoXIM·FoodPowder” product was created; it is a white powder of micrometric aggregates of hydroxyapatite suited for food products that cannot incorporate aqueous ingredients, like some dietary pills or specific types of gums [89].

As it can be seen from the presented results, the newly designed material, HApL showed significant antimicrobial properties against MRSA and *E. coli* ESBL bacterial strains. These results could significantly contribute to the development of new applications for cosmetic, pharmaceutical, medical or food industry. Their potential application in bone reconstruction could help reduce the number of postoperative infections after different implants. Moreover, the new materials obtained could play an important role in the food industry, and could be used to develop drugs that target bone health or dental hypersensitivity.

## 3. Materials and Methods 

Plant EOs used in this study were represented by lavender EO (*Lavandula angustifolia Sevastopolis)* and basil EO (*Ocimum basilicum* L.). In order to obtain the plant materials for oil extraction, *Ocimum basilicum* L. whole plants were harvested at the beginning of the flowering stage (first week of August, 2015) from an independent farm situated in southeast Romania. The *Lavandula angustifolia Sevastopolis* plants were also obtained from a local farmer from southeast Romania in the initial flowering stage at the beginning of June 2015. The plants were dried, packed in paper bags, and stored in a cool and dry place. The two plant EOs were obtained by steam distillation, which allows the reduction of polar compound loss [89]. The lavender and basil EOs prepared according to R. Chanamai et al. [90] were added to the appropriate amount of water (4% ratio). The resulting liquid mixture was stirred at room temperature for 1 h, and then used for DLS and antimicrobial studies.

The resulting hydroxyapatite (2 mL), after washing and redispersion in deionized water [91], was added to the mixture of plant EO and water (4% ratio). The resulting solutions were stirred for 24 h at room temperature, after which they were analyzed by SEM and DLS studies. The hydroxyapatite (2 mL), after washing and redispersion in deionized water, was also analyzed by SEM and Energy Dispersive Spectroscopy (EDAX). Furthermore, the hydroxyapatite, after washing and redispersion in deionized water, was centrifuged and then dried in an oven at 80 °C, then analyzed by XRD.

To determine the crystal phase and crystallite size of prepared HAp powder, a Bruker D8 Advance diffractometer (Bruker, Karlsruhe, Germany) with nickel filtered CuK𝛼 (λ = 1.5418 Å) radiation and a high efficiency one dimensional detector (Lynx Eye type, Bruker, Karlsruhe, Germany), operated in integration mode, were used,. The data were collected from the 2θ range 20°–70°, with a step of 0.02° and 34 s measuring time per step. The crystallite size (D) of the prepared HAp was calculated by the Scherrer’s equation:

*D* = Kλ/βcosθ

where, *K* is a dimensionless shape factor, with a value close to unity, λ is the wavelength of Cu-Kα radiations (*λ =* 1.5405 Å), *β* is full width at half maxima of the most intense peak (211) and θ is the Bragg angle (in degrees).

The morphology of the newly developed materials was investigated by scanning electron microscopy (SEM), using a HITACHI S4500 microscope (Tokyo, Japan) equipped with an energy dispersive X-ray attachment (EDAX) (Tokyo, Japan).

Dynamic Light Scattering (DLS) measurements were conducted using a SZ-100 Nanoparticle Analyzer (HORIBA, Ltd., Kyoto, Japan) at 25 ± 1 °C. All samples were diluted in distilled water before analysis. The size distribution was obtained from the intensity autocorrelation function by regularization analysis implemented in the software package. Hydrodynamic diameters were calculated using the Stokes-Einstein equation [63]. 

The chemical composition of the oil samples were analyzed using the gas chromatography (GC) technique, using a Perkin Elmer gas chromatographer (PerkinElmer Inc, Waltham, MA, USA) equipped with a flame ionization detector (FID) (PerkinElmer Inc, Waltham, MA, USA) with two stationary fused silica columns (60 m × 0.25 mm × 0.25 μm film coating and 30 m × 0.32 mm × 0.25 μm film thickness). The working conditions were as follows: injector temperature 250 °C; the column temperature was increased linearly from 40 to 260 °C, with a rate of approximately 4 °C per minute; the carrier gas was H_2_; the amount of injected samples dissolved in methylene chloride (CH_2_Cl_2_) was 1 μL; and the injection volume was set at 1 mL/min with a split ratio of 1:30. 

The GC/MS analysis of the essential oils was performed using a Pekin-Elmer Turbomass Quadrupole mass spectrometer (PerkinElmer Inc, Waltham, MA, USA). The spectrometer was fitted with a fused silica capillary column (60 m × 0.32 mm; 0.25 μm film coating). The operating conditions were the same as those described, or the analytical gas chromatography (GC). The mass spectrometry (MS) parameters were EI mode, with an ionization voltage of 70 eV. The compounds were identified using the using NIST and Wiley Registry 8 Edition mass database, n-alkalene (C9-C22) hydrocarbon series (Nile, Italy), and by comparing the mass spectra with MS literature [92,93,94]. The results of compound content were presented as relative area percent (%). The surface percentages obtained from the determination of standard chromatogram were used for the quantitative analysis. 

*Qualitative analysis of antimicrobial activity.* The antibacterial assays were performed using one reference bacterial strain: *E. coli* ATCC 25922, and three clinical isolates from the collection of microorganisms in the Microbiology Department (Faculty of Biology, University of Bucharest): *E. coli* ESBL 4493 (an extended spectrum beta-lactamase producing strain), *S. aureus* 1426, methicillin-resistant *S. aureus* (MRSA) 1144. Microbial suspensions of 1.5 × 10^8^ CFU/mL corresponding to a 0.5 McFarland density were prepared from 15 to 18 h solid cultures, obtained on TSA (tryptone soy agar). Standardized suspensions were inoculated onto Muller Hinton agar (MHA) plates by swabbing. The plant EOs were solubilized in DMSO (1:1 ratio). The HAp combinations, HApL and HApB, were also solubilized in DMSO at a final concentration of 10 mg/mL. From each of the solubilized material, a volume of 5 μL was placed directly, aseptically and distinctively onto the inoculated MHA plates. Agar plates were incubated at 37 °C for 18–24 h and inhibition zones formed were measured in mm [69,70,71,72]. A pure DMSO control was included with each test to ensure that microbial growth was not inhibited by DMSO itself. Tests were performed in duplicates.

*Determining the minimum inhibitory concentration (MIC) values and minimum bactericidal concentration (MBC) values.* The quantitative evaluation of antibacterial activities was performed using the microdilution broth method [69,95,96,97,98,99,100,101]. Each well of 96-well microtiter plate was aliquoted with 100 μL of Mueller-Hinton broth (MHB). One hundred microliters of MHB to the 12th well (sterility control), whereas MHB was added to 11th well (growth control). A volume of 100 μL of each plant EOs, HAp EOs combinations, and HAp dissolved in DMSO (as described above) were added to the first well, and a serial two-fold dilution was performed by transferring 100 μL of the suspension to the subsequent wells up to the 10th well; the final 100 μL of the suspension was discarded. Bacterial suspension of 1.5 × 10^8^ CFU/mL was prepared starting from 18 to 24 h solid cultures obtained on TSA and diluted, to achieve a final inoculum density of 3 × 10^7^ CFU and 3 × 10^5^ CFU/mL. After incubation of the inoculated 96 well plates at 37 °C for 18–24 h, the minimum inhibitory concentration (MIC) values and minimum bactericidal concentration (MBC) values were determined using a conventional plating method. The lowest concentrations of material tested which visibly inhibited growth and respectively determined 99.9% growth inhibition upon subculturing on MHA plates after overnight incubation at 37 °C were used as the MIC and MBC values respectively. The results of the antimicrobial assays using an inoculum with a density of 3 × 10^7^ CFU and 3 × 10^5^ CFU/mL were similar. 

*Flow cytometric assay for detection of antimicrobial action.* Fluorescence intensity, forward and side scatter values were determined with an Accuri C6 plus flow cytometer. The bacterial population was gated in the FSC-SSC dot plots referring to cell size and granularity. Evaluation of bacterial membrane potential was carried out with DiBAC_4_(3) dye (*bis*-(1,3-dibutylbarbituric acid) trimethineoxonol] (Invitrogen/Life Technologies, Carlsbad, CA, USA). The green fluorescence of the dye was detected in channel of fluorescence FITC. For flowcytometric analysis, after incubation at 37°C for 24 h, bacterial cells exposed to subinhibitory concentrations (½ × MICs) (100 μL) were transferred to 1.5 mL Eppendorf tubes and stained with membrane potential sensitive dye DiBAC_4_(3) (final concentration of 0.5 μg/mL) for 30 min at 37 °C, in the dark. Bacteria without treatment served as controls. The AccuriCFlow Plus software was used for data analysis.

## 4. Conclusions

Our studies presented in this work fall within the general endeavors to develop new materials with potential antimicrobial effects with applications in the food industry, pharmaceutical, or medical fields for the prevention and/or treatment of infectious diseases. The studies on the hydrodynamic diameter of basil and lavender EOs in water are presented for the first time. The average hydrodynamic diameters of basil and lavender EOs in water and basil and lavender EOs coated HAp nanoparticles were determined from the autocorrelation function. The DLS investigations showed that aggregation was minimal for all the studied samples. The morphology of the HAp nanoparticles coated with basil and lavender EOs determined by SEM studies indicated that the plant EOs coatings have influenced the size of the HAp nanoparticles, i.e., a decrease was observed from HAp to HApB and HApL. The antimicrobial studies showed very good antimicrobial activity for lavender EO, with MIC and MBC values of <0.1% for *E. coli* ESBL producing strain and 1.56% for MRSA. HAp coated with lavender EO displayed a significantly elevated antimicrobial effect, when compared with HAp alone, suggesting the potential use of HAp nanoparticles as carriers of low concentration of lavender EO with antibacterial properties. Flow cytometric measurements showed that at ½ × MICs, lavender EO, HApB and HApL disrupted the membrane potential of the treated bacteria, thus indicating a possible antimicrobial mechanism of action of the materials. The most significant diminution of growth inhibitory effect has been observed against *E. coli.* These findings could significantly contribute to the development of new antimicrobial strategies in the fight to combat infections following prosthetic implantation.

## Figures and Tables

**Table 1:** Chemical composition of *Ocimum basilicum* L. oils from southern Romania.

No.	Compound	% of Total
		*Ocimum basilicum* L.
1	Linalool	65.95
2	1,8 cineole	10.57
3	τ-cadinol	4.74
4	Eugenol	3.76
5	α-trans-bergamotene	2.35
6	α-terneol	1.04
7	Borneol	0.9
8	germacrene D	1.38
9	α-cadinene	1.02
Identified from total area		91.71

**Table 2:** Chemical composition of *Lavandula angustifolia Sevastopolis* oils from southern Romania.

No.	Compound	% of Total
		*Lavandula angustifolia*
1	Linalool	47.55
2	Borneol	8.52
3	Camphor	9.67
4	terpinene-4-ol	3.8
5	Myrcene	0.68
6	Camphene	0.56
7	α-pinene	0.54
8	Sabinene	0.5
9	Limonene	0.24
10	β-phellandrene	0.16
11	α-terpinene	0.06
12	1-8-cineole	8.6
13	linalool acetate	3.75
14	α-terpineol	1.35
15	Cryptone	1.25
16	geranyl acetate	0.98
Identified from total area		79.61

**Table 3:** Parameters comparison of mean particle size resulting from SEM and DLS analysis.

Sample	SEM (nm)	DLS (nm)
HAp	88.5 ± 3	273.86 ± 2
HApB	76.8 ± 5	265 ± 2
HApL	63.3 ± 6	257.76 ± 2

**Table 4:** Screening of antimicrobial activities of plant EOs, HApB and HApL respectively, via an adapted diffusion method against Gram-positive and Gram-negative bacteria.

Plant EOs and Plant EOs-HAp Combinations (Concentration)	Inhibition Zone (mm)					
	Lavander EO	HapL (10 mg/mL)	BasilEO	HApB (10 mg/mL)	Hap (10 mg/mL)	DMSO
Bacterial strain						
*E. coli* ATCC 25922	20 ± 1	15 ± 1	9 ± 2	7 ± 1	*-	*-
*E. coli* ESBL 4493	16 ± 0.5	10 ± 2	8 ± 1	6 ± 1	*-	*-
*S. aureus* 1426	25 ± 1	13 ± 2	10 ± 1	8 ± 1	*-	*-
MRSA 1144	24 ± 0.5	10 ± 2	7 ± 2	6 ± 1	*-	*-

* No inhibition zone observed.

**Table 5:** Minimum inhibitory concentration (MIC) values and minimum bactericidal concentration (MBC) values determined based on conventional plating method for Plant EOs.

Plant EOs Bacterial Strain	Lavander EO (1:1)		Basil EO (1:1)	
	MIC (%)	MBC (%)	MIC (%)	MBC (%)
*E. coli* ATCC 25922	<0.1	<0.1	25	40
*E. coli* ESBL 4493	0.19	0.19	30	45
*S. aureus* 1426	0.78	1.56	40	50
MRSA 1144	0.78	1.56	45	50

**Table 6:** Minimum inhibitory concentration (MIC) values and minimum bactericidal concentration (MBC) values determined based on conventional plating method for HAp, HApB and HApL.

Sample	HApL		HApB		HAp	
Bacterial Strain	MIC (mg/mL)	MBC (mg/mL)	MIC (mg/mL)	MBC (mg/mL)	MIC (mg/mL)	MBC (mg/mL)
*E. coli* ATCC 25922	0.15	0.31	5	5	5	5
*E. coli* ESBL 4493	0.62	0.62	>5	>5	>5	>5
*S. aureus* 1426	0.31	0.62	5	>5	5	>5
MRSA 1144	0.31	0.62	5	>5	5	>5
